# An Analysis of Artificial Reef Fish Community Structure along the Northwestern Gulf of Mexico Shelf: Potential Impacts of “Rigs-to-Reefs” Programs

**DOI:** 10.1371/journal.pone.0126354

**Published:** 2015-05-08

**Authors:** Matthew J. Ajemian, Jennifer J. Wetz, Brooke Shipley-Lozano, J. Dale Shively, Gregory W. Stunz

**Affiliations:** 1 Harte Research Institute for Gulf of Mexico Studies, Texas A&M University-Corpus Christi, Corpus Christi, Texas, United States of America; 2 Artificial Reef Program, Texas Parks and Wildlife Department, Austin, Texas, United States of America; North Carolina State University, UNITED STATES

## Abstract

Artificial structures are the dominant complex marine habitat type along the northwestern Gulf of Mexico (GOM) shelf. These habitats can consist of a variety of materials, but in this region are primarily comprised of active and reefed oil and gas platforms. Despite being established for several decades, the fish communities inhabiting these structures remain poorly investigated. Between 2012 and 2013 we assessed fish communities at 15 sites using remotely operated vehicles (ROVs). Fish assemblages were quantified from standing platforms and an array of artificial reef types (Liberty Ships and partially removed or toppled platforms) distributed over the Texas continental shelf. The depth gradient covered by the surveys (30–84 m) and variability in structure density and relief also permitted analyses of the effects of these characteristics on fish richness, diversity, and assemblage composition. ROVs captured a variety of species inhabiting these reefs from large transient piscivores to small herbivorous reef fishes. While structure type and relief were shown to influence species richness and community structure, major trends in species composition were largely explained by the bottom depth where these structures occurred. We observed a shift in fish communities and relatively high diversity at approximately 60 m bottom depth, confirming trends observed in previous studies of standing platforms. This depth was also correlated with some of the largest Red Snapper captured on supplementary vertical longline surveys. Our work indicates that managers of artificial reefing programs (e.g., Rigs-to-Reefs) in the GOM should carefully consider the ambient environmental conditions when designing reef sites. For the Texas continental shelf, reefing materials at a 50–60 m bottom depth can serve a dual purpose of enhancing diving experiences and providing the best potential habitat for relatively large Red Snapper.

## Introduction

Artificial reefs are distributed throughout the world’s oceans and serve a variety of purposes. These include increasing fisheries production, enhancing recreational opportunities such as fishing and diving, and creating new habitat for restoration and mitigation [1–5]. Interest in artificial reef development in the United States began in the 1950s and was primarily driven by recreational fishermen [6]. Concurrently, offshore oil and gas exploration was beginning in the Gulf of Mexico (GOM) with the first rigs installed off the state of Louisiana in the late 1940s. These artificial structures were quickly identified as prime fishing locations by fishermen throughout the GOM, and in 1979 the first offshore platform was removed from production off Louisiana and reefed in Florida [6–8]. By 1983, approximately 4000 oil and gas production platforms (hereafter referred to as “platforms”) had been put into place in the Gulf of Mexico, and although unintentional, these structures along with other reefed materials (e.g., ships, concrete bridge components, prefabricated concrete pyramids, etc.), made up the largest artificial reef complex in the world [9]. In response to growing public interest in reefing activity, the National Fishing Enhancement Act (NFEA) was passed in 1984. Policies set forth in NFEA led to the development of the National Artificial Reef Plan which helped guide the development of individual state reefing programs. These state-run Rigs-to-Reefs (RTR) programs, working with oil and gas operators, may choose to repurpose these structures as permitted artificial reefs. As of 2012, approximately 420 platforms have been reefed under state artificial reef plans, with Louisiana and Texas having the largest RTR programs (www.bsee.gov). As a large portion of existing oil and gas platforms are reaching their production lifespans, the amount of material available to state reefing programs is growing. In 2012, there were approximately 3000 platforms in the GOM and 359 of those were expected to be decommissioned by the end of 2013.

With a growing source of material for RTR programs worldwide, recent debates have highlighted questions related to the productivity and habitat function of artificial structures. Numerous studies have documented increases in fish (adult, larval and juvenile) abundance and recruitment to platforms and other artificial structures [3, 10–13], and catch rates of fisheries species can also increase over and around these reefs [14–16]. However, the source (redistribution, aggregation or actual stock enhancement) of the increase is still debated in the literature [2, 5, 10, 17–19]. For oil and gas platforms off the California coast, scientists have suggested that both the upper depths and the deeper structure may be important to certain species at various life-stages, with the platforms essentially acting as both recruitment habitat for juvenile fish and possible refuge areas for adults [8, 20–22]. In addition, a recent analysis by Claisse et al. [23] estimated secondary fish production at oil and gas platforms off the coast of California to be substantially greater than any other marine ecosystem. This work, in conjunction with trophic analyses in the Mediterranean’s largest artificial reef system [24], indicates that non-natural habitats can indeed substantially contribute to local secondary production.

Studies of GOM platforms have documented much of the biomass difference associated with depth and the area of influence of these structures [25–28]. Other studies have compared natural hard-bottom areas to the artificial structure provided by platforms [29–31], and have shown that the fish community composition is different between these two types of structure. Diversity is greater at natural sites, while biomass per unit area is greater at standing platforms [29, 30]. For the most economically important species in the GOM, Red Snapper (*Lutjanus campechanus*), Wilson et al [29] noted that this species was proportionally most abundant at a low-relief natural hard-bottom area. However, it has been suggested that the addition of artificial habitat has resulted in an increase in the harvest potential of Red Snapper and any decrease in artificial structure (such as large-scale platform removals) may have negative results on these populations [19]. Recent evidence also suggests that some artificial structures in the GOM may serve as long-term residence sites for reef-associated species such as Red Snapper [32, 33]. While it appears that larger (age 3+) Red Snapper may migrate to deeper, less vertically structured habitat, oil and gas platforms may harbor greater numbers of younger age-2 Red Snapper [10]. Off the south Texas coast, where natural reefs and platforms are limited in number, removal of existing platforms may affect reef fish populations and could limit settlement of reef fishes [11]. Because the number of platforms has been predicted to decline 29% (or more) from 1999–2023 as removals exceed installations [34], additional studies that compare the habitat provided by standing and reefed oil and gas platforms to reef fishes are especially important and timely. Although RTR options do not include retaining the full vertical extent of the structure (i.e. standing platforms extending out of water), an evaluation of how the community may change with removal of the upper portion (from surface down to 50ft and deeper) remains important for estimating the potential impacts of these programs. For economically important and heavily managed fish species, such as Red Snapper, an evaluation of possible reef orientations (i.e. partial removals, toppled) and placement (water depth, distance from shore, number of structures per reef site) is also critical, yet poorly investigated in the scientific literature. For instance, Strelcheck et al. [35] found a negative correlation between reef fish biomass and artificial reef abundance. Mudrak and Szedlmayer [36] also suggest that artificial reefs intended to harbor younger fish should not be placed in close proximity to structures such as natural hardbottom that are typically inhabited by larger adult fish.

It appears that both structure type and location may play a role in determining the fish community at artificial reef sites, and to establish the best management practices for RTR programs, these questions clearly warrant further exploration. The only recent comparison of RTR options in the GOM was a study conducted by Wilson et al. [30]. While this work was seminal and informative, it drew comparisons from a relatively small set of sites and may not be representative of the entire region. Recent reviews on RTR controversy indicate the need for science-based decision making in relation to the proper use of these structures [37–40]. In other regions of the US and the world, many groups look to policies in the Gulf of Mexico to help influence decisions related to decommissioning options elsewhere.

The overall goal of this study was to assess artificial reef fish communities in the western GOM using remotely operated vehicles (ROVs). Fish assemblages were described and quantified from an array of artificial reef types (Liberty Ships, partially removed and toppled platforms) and active standing platforms broadly distributed over the Texas continental shelf. The depth gradient covered by the surveys and variability in structure density and relief also permitted analyses into the effects of these characteristics on artificial reef fish assemblages. Lastly, using fishery-independent data on Red Snapper abundance and size across these artificial reefs, we examined potential regions that could contribute to re-building stocks of this critical fishery in the GOM.

## Methods

### Ethics Statement

This study was carried out under a specific protocol ("South Texas Artificial Reef Monitoring—Fish Community Surveys; #04–12) approved by the Institutional Animal Care and Use Committee at Texas A&M University-Corpus Christi. All efforts were made to minimize animal suffering during collection. Sampling occurred in both state and federal waters under a permit issued by Texas Parks and Wildlife Department (SPR-0303-279) and Letters of Acknowledgement from the National Marine Fisheries Service. All sampling procedures were reviewed or specifically approved as part of obtaining the field permit and Letter of Acknowledgement.

### Artificial Reef Site Description and Location

**Fig 1:**
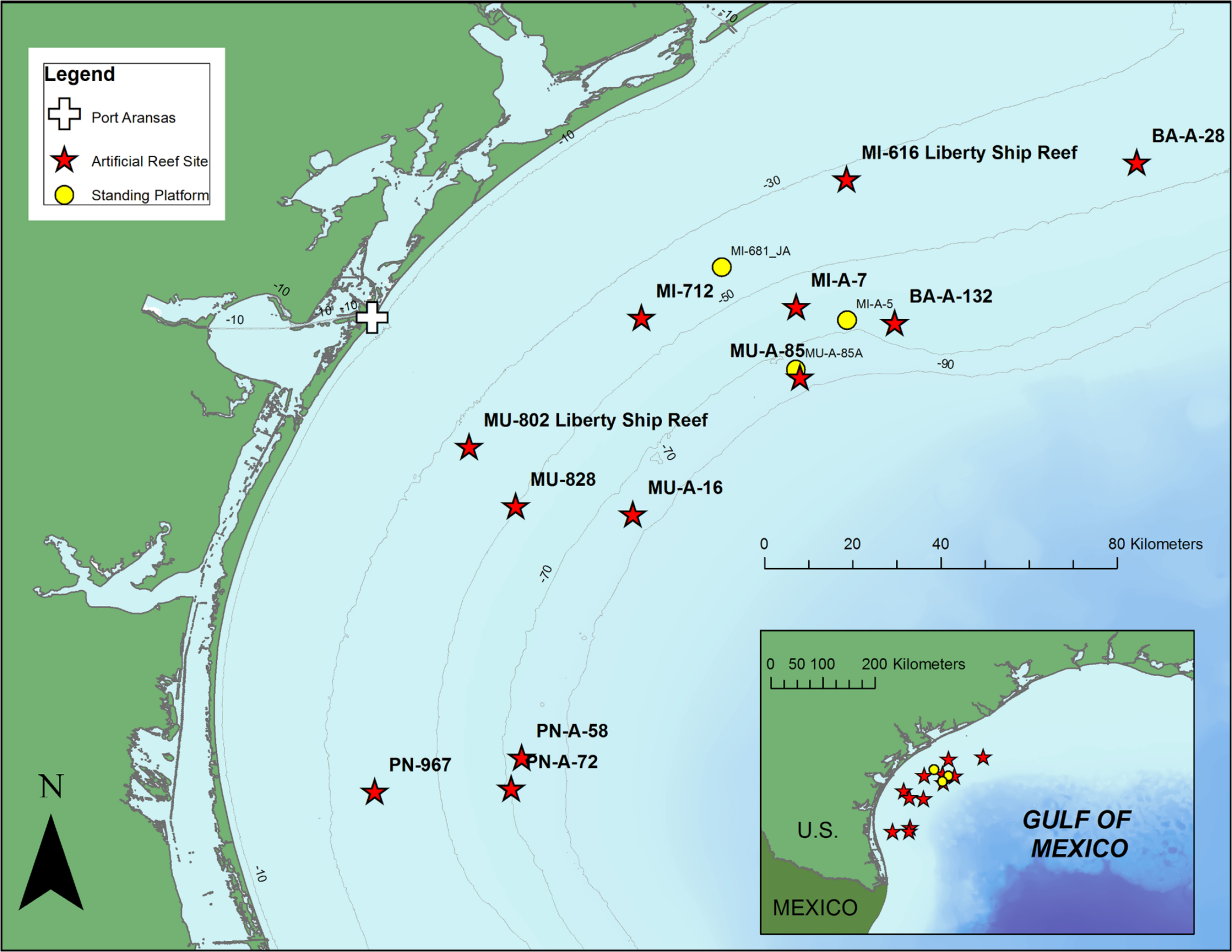
Map of ROV Survey Sites. Artificial reef sites (red stars) and platforms (yellow circles) surveyed with ROVs between 2012 and 2013. Bathymetric countours are indicated by gray lines in 20 m increments. Inset map indicates location of survey sites relative to Gulf of Mexico.

Our study region included 12 artificial reef sites and 3 standing platforms situated along the Texas coast in shelf waters of the northwestern Gulf of Mexico (Fig 1). Multiple structures of varying materials were reefed within each artificial reef site. The predominant bottom type surrounding the reefs was a silt-clay mixture throughout the study area. A total of 44 ROV surveys were completed on various artificial structures in 2012 and 2013 (Table 1). These surveys included standing oil and gas platforms (*n* = 5), platforms jackets that were partially removed or “cutoff” (*n* = 14) or toppled (*n* = 14), a platform deck (n = 2), and Liberty Ships (*n* = 9). All oil and gas platform materials (toppled and partially removed jackets) are hereafter referred to as “Rigs-to-Reefs” structures. Vertical relief (top of structure to benthos) was variable among structure types with standing platforms having the highest relief (40–79 m; $\bar{x}$ = 62 m), followed by partial removals (19–56 m; $\bar{x}$ = 36 m), toppled platforms (10–39 m; $\bar{x}$ = 19 m), the deck (13 m), and Liberty Ships (5–9 m; $\bar{x}$ = 7 m).

**Table 1:** Structural characteristics of artificial reefs and standing platforms surveyed between 2012 and 2013.

Site	Structure Material	Structure Type	Number of Structures	Survey Date	ROV	Structure Height	Bottom Depth
MU-802	ship	ship	3	4/10/2012	VideoRay	5	34
MI-681-JA*	4-pile platform	standing	1	5/22/2012	VideoRay	40	40
MI-A-5*	3-pile platform	standing	1	5/22/2012	VideoRay	67	67
MU-A-85A*	8-pile platform	standing	1	5/22/2012	VideoRay	79	79
MI-712	Deck	deck	3	6/7/2012	VideoRay	13	40
MU-828	4-pile jacket	topple	8	6/7/2012	VideoRay	20	50
MI-A-7	4-pile jacket base	cutoff	12	6/8/2012	VideoRay	33	60
MU-A-85A*	8-pile platform	standing	1	6/26/2012	VideoRay	79	79
MU-A-16	8-pile jacket base	topple	6	9/21/2012	VideoRay	18	83
MU-A-16	8-pile jacket top	cutoff	6	9/21/2012	VideoRay	56	83
MU-A-85	8-pile jacket base	cutoff	3	9/21/2012	VideoRay	55	84
MU-A-85	4-pile jacket	cutoff	3	9/21/2012	VideoRay	23	84
MU-A-85A*	8-pile platform	standing	1	9/21/2012	VideoRay	79	79
BA-A-28	4-pile jacket	topple	4	10/9/2012	OceanEx	14	46
BA-A-28	4-pile jacket	cutoff	4	10/9/2012	OceanEx	19	46
MU-802	ship	ship	3	10/12/2012	VideoRay	5	34
PN-967	4-pile jacket	topple	2	10/15/2012	OceanEx	10	36
PN-967	4-pile jacket	topple	2	10/15/2012	OceanEx	16	36
PN-A-58	4-pile jacket base	cutoff	2	10/15/2012	OceanEx	23	75
PN-A-58	4-pile jacket top	topple	2	10/15/2012	OceanEx	15	75
PN-A-72	3-pile jacket base	cutoff	2	10/15/2012	OceanEx	40	72
PN-A-72	3-pile jacket top	topple	2	10/15/2012	OceanEx	39	71
BA-A-132	4-pile jacket	topple	4	10/16/2012	OceanEx	20	61
BA-A-132	8-pile jacket	topple	4	10/16/2012	OceanEx	29	61
MI-616	ship	ship	3	10/17/2012	VideoRay	7	36
MI-616	ship	ship	3	10/17/2012	VideoRay	9	36
MU-828	8-pile jacket	topple	8	7/3/2013	VideoRay	20	50
MU-A-16	8-pile jacket base	cutoff	6	7/3/2013	VideoRay	56	83
MU-802	ship	ship	3	8/1/2013	VideoRay	5	34
MU-802	ship	ship	3	8/1/2013	VideoRay	5	34
MI-616	ship	ship	3	8/9/2013	VideoRay	5	36
MI-616	ship	ship	3	8/9/2013	VideoRay	7	36
MI-712	Deck	deck	3	8/9/2013	VideoRay	13	40
MI-712	Barge	ship	3	8/9/2013	VideoRay	3	40
MI-A-7	4-pile jacket top	cutoff	12	8/10/2013	VideoRay	34	60
PN-967	4-pile jacket	topple	2	8/22/2013	VideoRay	10	36
PN-967	4-pile jacket	topple	2	8/22/2013	VideoRay	16	36
PN-A-58	4-pile jacket base	cutoff	2	8/22/2013	VideoRay	23	75
PN-A-72	3-pile jacket base	cutoff	2	8/22/2013	VideoRay	40	72
BA-A-132	8-pile jacket	topple	4	9/25/2013	VideoRay	29	61
MU-A-85	8-pile jacket base	cutoff	3	9/25/2013	VideoRay	55	84
BA-A-132	8-pile jacket	topple	4	10/8/2013	VideoRay	29	61
MI-A-7	4-pile jacket top	cutoff	12	10/8/2013	VideoRay	34	60
MU-A-85	8-pile jacket base	cutoff	3	10/8/2013	VideoRay	55	84

Asterisks (*) denote standing platforms.

### ROV Surveys of Fish Communities

We used a VideoRay Pro 4 micro-ROV equipped with a compass, depth sensor, temperature sensor, auto-depth holding capabilities, forward facing color camera (520 line, 0.1 lux), LED array for illumination, LYNN Hawk Video enhancer software to enhance video in poor visibility, and laser scaler to estimate fish size (8 cm between lasers). The ROV was piloted with an integrated control box connected via a tether. Surface real-time observations were conducted with live feed from the camera (160° tilt and a 105° viewing angle). Depth and heading were visible on the real-time image screen. Because the VideoRay Pro 4 system did not record high-definition footage, we additionally mounted a GoPro camera (HD Hero2). The HD Hero2 filmed at 960p (30 fps) and had a 170° field of view. However, because GoPro cameras had restricted use and battery life, footage from these devices was used to solely supplement identification, with all counts conducted within the VideoRay field of view. The VideoRay system was used for a total of 34 dives. Our study also used a larger working-class ROV, the Global Explorer (Deep Sea Systems International, Inc.), to complete 10 surveys of artificial reefs during an oceanographic cruise aboard the R/V Falkor (8–20 October 2012). The Global Explorer is a large (25,000-lb) deep water (3000-m rating) ROV, equipped with Ocean ProHD Cameras (1080i resolution), a digital photo and laser scaler, digital scanning sonar (BlueView), 2 vertical thrusters, 4 horizontal thrusters, and LED lights. Despite significant differences in the VideoRay and Global Explorer ROV size and capabilities, we conducted surveys of artificial reefs using the same standardized methods.

Continuous roving transect (CRT) methods were used to capture fish communities associated with artificial reefs [41]. Where possible, we attempted to survey at least two structures per reefing site on a given survey day in 2012 and 2013, resulting in 3 surveys per site in most locations over the two years and at least 5 surveys per structure type.

Videos from the ROV recording systems (ROV standard, GoPro HD, and OceanPro HD) were downloaded to a computer and analyzed with open-source video software (VLC media player) in the laboratory. Fish were identified to the lowest possible taxon, enumerated and recorded onto a spreadsheet each time they entered the field of view. Time of day, depth of occurrence, temperature and heading of ROV, and the time in and out of the water (used to calculate a dive time) were recorded. We generated a MinCount for each species, which is the greatest number of individuals captured at any one time on the video. This conservative count represented the total number, at minimum, of individuals for a particular species during the dive and is the commonly preferred abundance metric reported for video survey data [41–45]. For each dive we also calculated species richness (i.e., total number of species observed), a Shannon-Weiner Diversity Index, and Evenness.

#### Univariate Analysis

We used analyses of variance (ANOVA) to assess the impact of structure type (cutoff, deck, standing, ship, topple) on overall fish diversity metrics. Analyses of variance (or Kruskal-Wallis for non-parametric data sets) were run on Species Richness, Shannon-Weiner Diversity Indices (H′) and Evenness (J). For tests with significant main effects, we ran Tukey’s pairwise comparisons to identify potential sources of variation and non-linear regression techniques to examine relationships between bottom depth and structure relief with species richness. Data were checked for normality and homogeneity of variance assumptions prior to all analyses. All ROV univariate analyses and curve fitting were conducted in SigmaPlot 12.0.

#### Multivariate Analysis

Overall sample size sufficiency (i.e., number of surveys) of fish assemblage data was assessed with a species accumulation curve. Curves were created in PRIMER v6 and estimated the cumulative number of fish species across samples (*S*_*obs*_) based on richness data from all sites surveyed. To remove the effect of sampling chronology on curve smoothness, the order was randomized across 999 permutations. If the curve approached an asymptote, the number of dives was considered sufficient in explaining fish diversity across our sites.

Fish community assemblage patterns were analyzed using multivariate methods. For each survey (*n* = 44) species-specific minimum counts were 4th-root transformed to develop a Bray-Curtis dissimilarity matrix. Non-metric multidimensional scaling (NMDS) was run on the matrix to visually assess the dispersion of samples and clustering by various factors. Assemblage differences were assessed with a one-way Analysis of Similarity (ANOSIM) using structure type as the factor and transformed species abundance (i.e., MinCount) data as the response variable. Pairwise comparisons were conducted for significant factors (α < 0.05) to determine the source of fish assemblage variation. We also employed a similarity of percent contribution (SIMPER) analysis to identify the species driving the disparity among various levels of structure type.

Because our fish assemblage data set included samples from a wide range of depths and structure dimensions, we followed our ANOSIM and SIMPER with additional analyses to identify additional potential drivers of variation in the fish assemblages among artificial reefs. Abiotic data (ambient bottom depth, structure relief, shallowest structure depth, and the number of reefed structures on site) were normalized and used to build a Euclidean distance-based resemblance matrix. These data were exposed to a non-parametric form of a Mantel test, RELATE, to assess agreement in the multivariate pattern between the biological and environmental resemblance matrices using a suite of random permutations. Following RELATE, we used a BEST analysis (i.e., Bio-env) to find the best match between multivariate among-sample patterns of fish assemblages and reef site characteristics (i.e., highest Spearman rank correlation value). We then used a CLUSTER analysis to visually identify similarities in fish communities among the various sites, and potential groupings by abiotic factors. All community and multivariate analyses were conducted using Primer Version 6.0 [46].

### Vertical Longline Surveys for Red Snapper

Given the visibility constraints on the lower portion of the water column at some locales (i.e., benthic nepheloid layer) and preference for these habitats by Red Snapper [41, 47], we supplemented our ROV methods with opportunistic Vertical Longline (VLL) surveys to estimate the abundance and size of Red Snapper across artificial structures. The VLL surveys used the published Southeast Area Monitoring and Assessment Program (SEAMAP) protocol developed by researchers studying artificial reefs elsewhere in the Gulf of Mexico [48]. At each site we fished a backbone of 10 hooks at 3 haphazardly chosen locations 5–20 m from the edge of the reef perimeter. Each “set” consisted of a drop of three hook sizes (8/0, 11/0, and 15/0 stainless steel circle hooks) and was fished at a stationary position along bottom for 5 minutes. A total of 126 vertical line sets were made across various artificial structure types (topple = 22, standing = 41, cutoff = 30, ship = 16, deck = 8, and concrete = 9) between 2012 and 2014. All fish captured were measured (SL, FL, and TL), weighed, and either tagged and released or kept for other biological studies.

Red Snapper catch per unit effort (CPUE; number of fish caught per set), biomass per unit effort (total weight per set), and mean total length were compared among the structure types fished with ANOVA. The CPUE from nearshore concrete reefs was not included in ANOVA tests given the significantly younger age of the reef (<1 yr) compared to other sites. These metrics were also plotted with bottom depth to explore potential spatial trends in Red Snapper abundance and size with the full data set.

## Results

### ROV Surveys

#### Overall Trends

**Fig 2:**
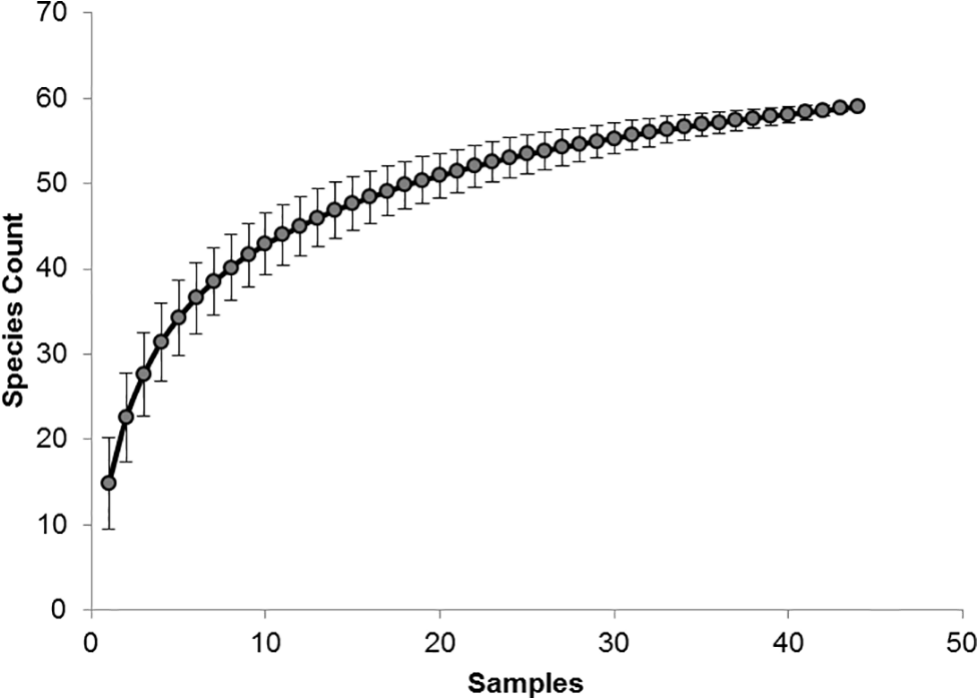
Species-Accumulation Plot. Randomly permutated species accumulation curve based on fish assemblages observed from remotely operated vehicle surveys. (Michaelis-Menton *S*_max_ = 61.12).

Analysis of two years of ROV surveys showed sufficient sampling effort to explain overall fish species diversity across artificial reef sites. The randomly permutated species accumulation curve approached an asymptote (Michaelis-Menton *S*_*max*_ = 61.16) indicating the sample size captured an estimated 96% of the fish species discernible from our ROV surveys (Fig 2). A total of 59 fish species were identified from the video footage, representing 19 families (Table 2). This assemblage included fishes from a variety of sizes from large (est. > 2 m) carcharhinid sharks (*Carcharhinus plumbeus*, *C*. *falcliformis*) to small pomacentrid damselfishes (est. <10 cm). The highest number of species identified at one site (including all surveys) was 35 at BA-A-132, a toppled platform. The least species-rich site was a Liberty Ship site (MU-802) with 14.

**Table 2:** Locations where various taxa were recorded on remotely operated vehicle surveys between 2012 and 2013.

Family	Common Name	Scientific Name	MU-802	MI-616	PN-967	MI-681-JA*	MI-712	BA-A-28	MU-828	MI-A-7	BA-A-132	MI-A-5*	PN-A-72	PN-A-58	MU-A-85A*	MU-A-16	MU-A-85
Acanthuridae	Blue Tang	*Acanthurus coeruleus*								X	X				X	X	X
	Doctorfish	*Acanthurus chirurgus*									X						
Balistidae	Gray Triggerfish	*Balistes capriscus*	X	X	X	X	X	X	X	X							
	Ocean Triggerfish	*Canthidermis sufflamen*													X		
Carangidae	African Pompano	*Alectis ciliaris*			X				X	X	X						
	Almaco Jack	*Seriola rivoliana*			X	X	X	X		X	X			X	X	X	X
	Bar Jack	*Caranx ruber*		X			X	X				X			X	X	
	Black Jack	*Caranx lugubris*		X							X			X			X
	Blue Runner	*Caranx crysos*	X	X	X		X	X	X	X		X	X		X	X	
	Crevalle Jack	*Caranx hippos*			X	X	X			X	X	X	X	X	X		X
	Greater Amberjack	*Seriola dumerili*				X		X	X	X	X	X	X	X	X	X	X
	Horse-eye Jack	*Caranx latus*	X		X		X	X		X	X	X	X	X	X	X	X
	Lookdown	*Selene vomer*	X	X	X	X	X		X	X		X	X	X		X	
	Palometa	*Trachinotus goodei*			X												
	Permit	*Trachinotus falcatus*									X						
	Rainbow Runner	*Elagatis bipinnulata*		X	X			X		X			X	X			X
	Yellow Jack	*Caranx bartholomaei*	X	X	X		X	X	X	X	X				X	X	X
Carcharhinidae	Sandbar Shark	*Carcharhinus plumbeus*								X	X	X				X	X
	Silky Shark	*Carcharhinus falciformus*															X
Chaetodontidae	Reef Butterflyfish	*Chaetodon sedentarius*			X					X	X			X			X
	Spotfin Butterflyfish	*Chaetodon ocellatus*			X		X	X		X	X			X	X		
Dasyatidae	Southern Stingray	*Dasyatis americana*		X													
Ephippidae	Atlantic Spadefish	*Chaetodipterus faber*	X	X	X	X	X	X	X	X			X	X			
Haemulidae	Black Margate	*Anisotremus surinamensis*	X		X		X										
	Porkfish	*Anisotremus virginicus*			X		X										
	Tomtate	*Haemulon aurolineatum*		X	X		X	X			X						
Holocentridae	Squirrelfish	*Holocentrus adscensionis*			X			X			X						
Kyphosidae	Bermuda Chub	*Kyphosus sectatrix*				X	X	X		X		X			X	X	X
Labridae	Bluehead wrasse	*Thalassoma bifasciatum*								X	X	X		X	X	X	X
	Creole wrasse	*Clepticus parrae*									X						X
	Spanish Hogfish	*Bodianus rufus*	X	X	X	X	X	X	X	X	X	X	X	X	X	X	X
	Spotfin Hogfish	*Bodianus pulchellus*			X	X	X	X	X	X	X		X	X	X	X	X
Lutjanidae	Cubera Snapper	*Lutjanus cyanopterus*									X						
	Dog Snapper	*Lutjanus jocu*													X		
	Gray Snapper	*Lutjanus griseus*	X	X	X	X	X	X	X	X	X		X	X	X	X	X
	Lane Snapper	*Lutjanus synagris*	X														
	Red Snapper	*Lutjanus campechanus*	X	X	X	X	X	X	X	X	X	X	X	X	X	X	X
	Vermillion Snapper	*Rhomboplites aurorubens*			X		X	X	X	X	X		X	X	X		X
	Yellowtail Snapper	*Ochyurus chrysurus*			X												
Pomacanthidae	Blue Angelfish	*Holacanthus bermudensis*	X	X	X		X	X	X	X	X		X	X		X	X
	French Angelfish	*Pomacanthus paru*						X		X	X				X		X
	Rock Beauty	*Holacanthus tricolor*															X
	Queen Angelfish	*Holacanthus ciliaris*					X	X		X			X	X	X		
	Townsend Angelfish	*Holacanthus* sp.						X			X			X			
Pomacentridae	Bicolor Damselfish	*Stegastes partitus*												X	X		
	Brown Chromis	*Chromis multilineata*									X	X			X		
	Damselfish sp.	*Stegastes sp*.		X	X				X	X	X		X	X		X	X
	Sergeant Major	*Abudefduf saxatilis*							X	X		X			X		X
	Yellowtail Damselfish	*Microspathodon chrysurus*													X		
Rachycentridae	Cobia	*Rachycentron canadum*			X					X			X	X			
Serranidae	Creole Fish	*Paranthias furcifer*			X		X	X		X	X		X	X	X	X	X
	Goliath Grouper	*Epinephelus itajara*			X												
	Rock Hind	*Epinephelus adscensionis*	X	X	X	X	X	X	X	X	X	X	X	X	X	X	X
	Scamp	*Mycteroperca phenax*									X	X		X			
	Yellowmouth Grouper	*Mycteroperca interstitialis*									X						
Sparidae	Sheepshead	*Archosargus probatocephalus*	X	X	X	X	X	X	X								
Sphyraenidae	Great Barracuda	*Sphyraena barracuda*			X	X	X	X	X	X	X	X	X	X	X	X	X
Scorpaenidae	Lionfish	*Pterois volitans*							X	X	X					X	
Tetradontidae	Sharpnose Puffer	*Canthigaster rostrata*									X			X			
**Number of species per site**		** **	**14**	**17**	**31**	**14**	**25**	**26**	**19**	**32**	**35**	**16**	**19**	**27**	**27**	**21**	**27**
**Number of site visits 2012–2013**		** **	**2**	**2**	**2**	**1**	**2**	**1**	**2**	**3**	**3**	**1**	**2**	**2**	**3**	**2**	**3**
**Bottom Depth of site (m)**			**34**	**36**	**36**	**40**	**40**	**46**	**50**	**60**	**61**	**67**	**72**	**75**	**79**	**83**	**84**

Taxa included are only those that could be identified to genus level. Sites are sorted from shallow to deep, from left to right. Standing platform sites are marked with an asterisk.

Species most commonly sighted (at 100% of sites surveyed) included Spanish Hogfish (*Bodianus rufus*), Gray Snapper (*Lutjanus griseus*), Red Snapper (*Lutjanus campechanus*), and Rock Hind (*Epinephelus adscensionis*) (Table 2). Also commonly present (>80% of sites surveyed) were Great Barracuda (*Sphraena barracuda*), Blue Angelfish (*Holocanthus bermudensis*), Horse-eye Jack (*Caranx latus*), and Spotfin Hogfish (*Bodianus pulchellus*). Fish indices of abundance (based on MinCount) were summarized by species for each ROV survey.

Lutjanid species, including Red, Gray, and Vermillion snappers accounted for 26% of the total counts at these sites for both years. A large portion of total fish abundance was represented by pelagic species such as Horse-eye Jack (*Caranx latus;* 13%), Blue Runner (*Caranx crysos;* 12%), and Lookdown (*Selene vomer;* 6%).

**Fig 3:**
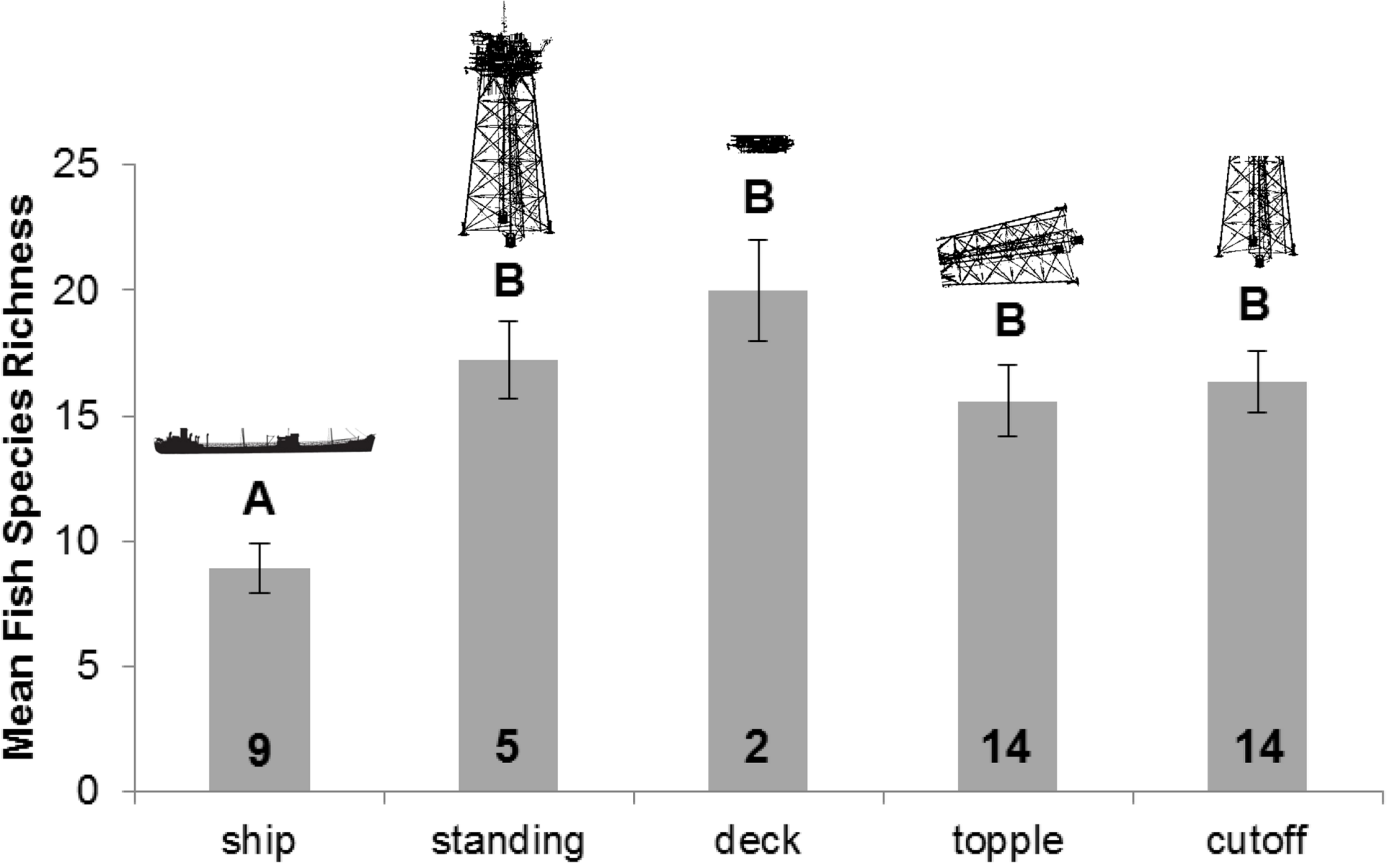
Vertical bar chart of mean species richness by structure type. Sample size (i.e., number of surveys) is represented by the number at the bottom of each bar. Error bars represent standard errors. Letter designations (A, B) correspond to statistically different groups based on Tukey’s pair-wise comparisons tests (α = 0.05).

Species richness was significantly affected by structure type (One-way ANOVA, *F*_*4*,*43*_ = 5.578; *P* = 0.001) with mean richness significantly lower on Liberty Ships than all other structure types (Fig 3). There were no observed impacts of structure type on Shannon-Weiner Diversity indices (One-way ANOVA, *F*_*4*,*43*_ = 2.183; *P* = 0.089), although statistical test power was below the desired level. Structure type also significantly affected maximum diversity (One-way ANOVA, *F*_*4*,*43*_ = 7.641; *P* < 0.001), with ships having significantly lower values than all other structure types. There was no significant effect of structure type on evenness (One-way ANOVA, *F*_*4*,*43*_ = 0.978; *P* = 0.431).

**Fig 4:**
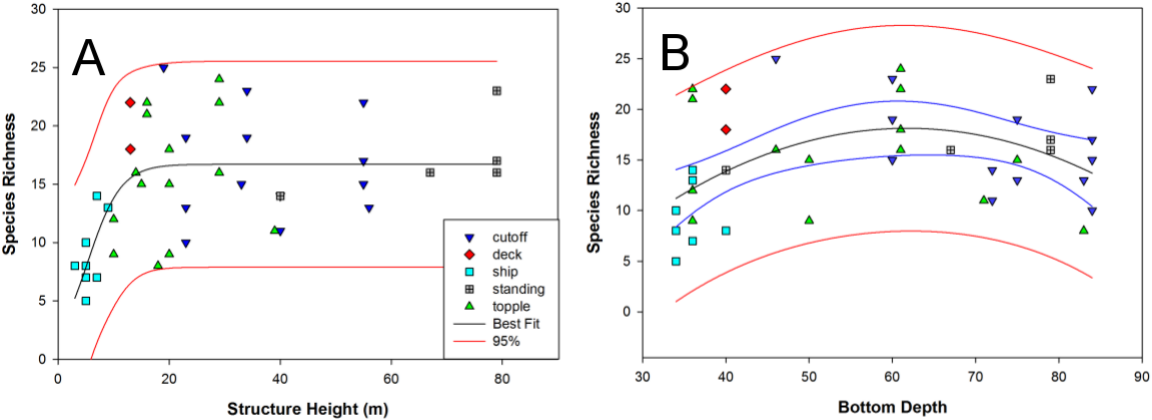
Scatter plots of species richness by structure height (A) and bottom depth (B). Various structure types are categorized by symbol and color. Non-linear curve fits are superimposed on each plot with best fit (black line) and 95% confidence bands (red line).

Scatterplots of species richness indicated a non-linear relationship with structure height (i.e., relief; Fig 4A). Curve-fitting data from all structure types showed these two variables significantly fit a sigmoidal curve (*F*_2,44_ = 12.32, *P* < 0.0001; *R* ^2^ = 0.38), suggesting that species richness saturated at a structure height of approximately 20 m. Fish species richness exhibited a weaker (*R* ^2^ = 0.20), though statistically significant quadratic relationship with bottom depth (*F*_2,44_ = 5.1572, *P* = 0.01), where richness was slightly more elevated at mid-depth sites (i.e., 60 m) compared to shallow and deep sites (Fig 4B).

#### Spatial and Temporal Trends in Managed Species

**Fig 5:**
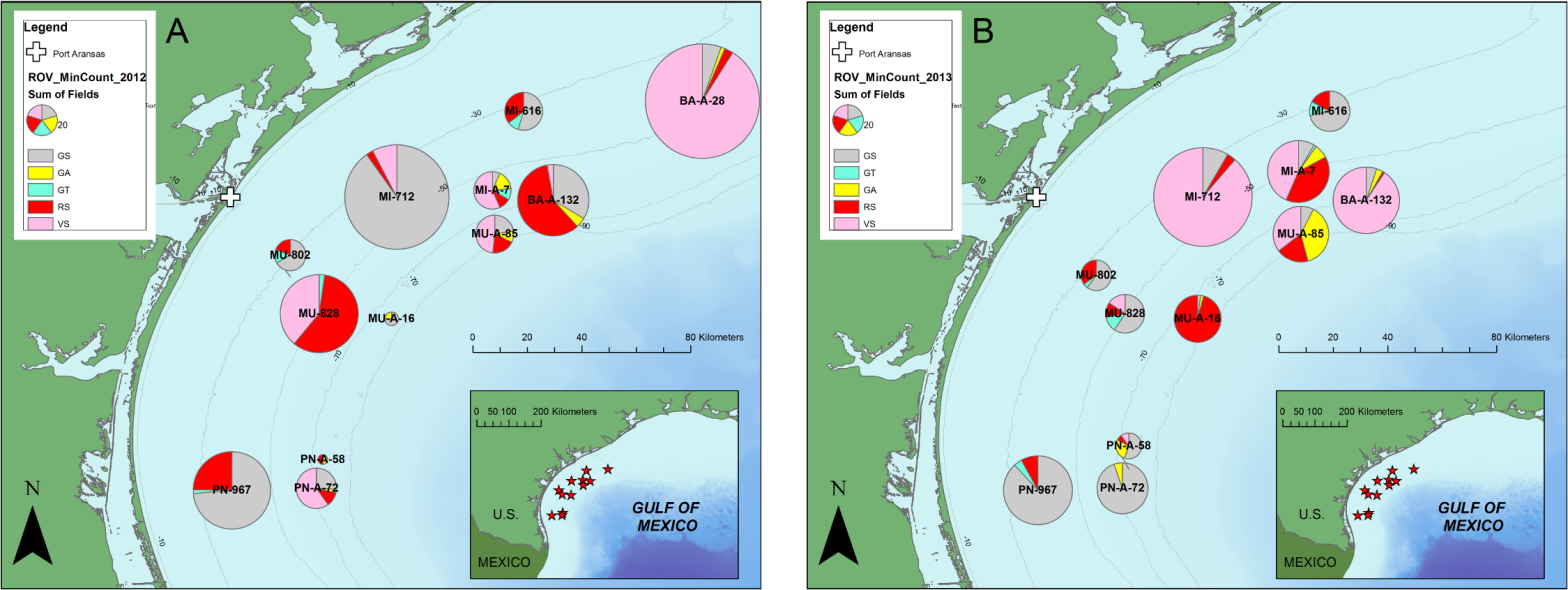
Map of pie charts depicting the indices of abundance of five federally managed species. Data are shown separately for 2012 (A) and 2013 (B): GS = Gray Snapper (gray), GA = Greater Amberjack (yellow), GT = Gray Triggerfish (cyan), RS = Red Snapper (red), and VS = Vermilion Snapper (pink).

Minimum counts for common federally managed species exhibited high variability among sites and years for all species (Table 3, Fig 5A and 5B). In 2012, the highest Red Snapper MinCount (77) was observed along a toppled platform (MU-828), whereas the highest MinCount in 2013 (46) was seen at MU-A-16, a cutoff platform. Vermillion Snapper minimum counts ranged even more widely, with the highest observed at site BA-A-28 in 2012 (255), and MI-712 (184) in 2013. Gray Triggerfish minimum counts never exceeded 4, and were also variable between years. Greater Amberjack were observed as many as 151 individuals at a time (MU-A-85, 2013), but similarly exhibited strong year-to-year variability.

**Table 3:** Abundance indices (i.e., minimum counts) from ROV surveys for five federally managed fisheries species in the Gulf of Mexico.

	ship (n = 9)		deck (n = 2)		cutoff (n = 14)		topple (n = 14)		standing (n = 5)	
Common Name	2012	2013	2012	2013	2012	2013	2012	2013	2012	2013*
										
Gray Snapper	17	25	211	18	15	53	95	89	35	N/A
Gray Triggerfish	3	4	1	1	3	1	3	4	4	N/A
Greater Amberjack	0	0	0	0	5	26	5	151	15	N/A
Red Snapper	11	7	5	2	6	46	77	8	46	N/A
Vermillion Snapper	0	2	18	184	255	36	51	86	28	N/A

For each structure type category, the highest counts are listed from all surveys of that structure type within a given year.

*—standing platforms could not be surveyed in 2013 due to logistics.

### Multivariate Analyses

**Fig 6:**
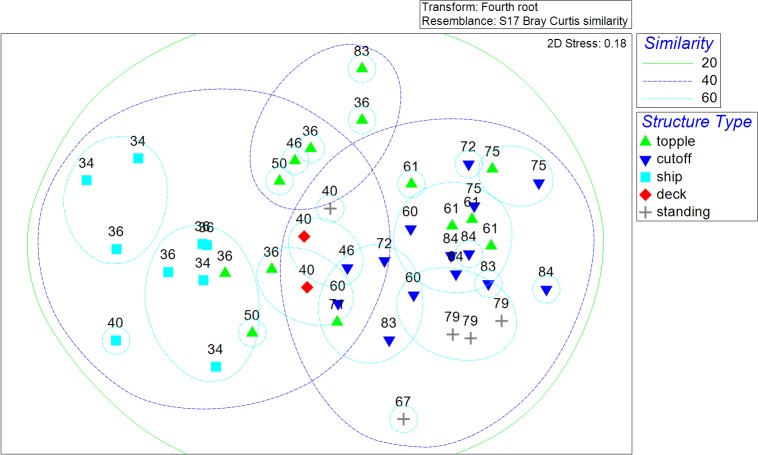
Non-metric multidimensional scaling (NMDS) plot of fish assemblages documented by remotely operated vehicle surveys. Bottom depths over overlain on symbols, with types and colors representing various structure types: topple (green triangle), cutoff (blue triangle), ship (blue square), deck (red diamond), and standing platform (gray cross). Percent similarity bubbles (from CLUSTER) are overlain at 20, 40, and 60%.

The NMDS ordination plot revealed significant clustering of fish community assemblages by structure type (Fig 6). The one-way ANOSIM showed that structure type significantly influenced fish assemblages (Global R = 0.434, P = 0.001). Subsequent pairwise comparisons were significant between most structure types with the exception of topple and deck (R = -0.252, P = 0.967) as well as standing and cutoff (R = 0.173; P = 0.09) (Table 4). Subsequent SIMPER analysis indicated that standing platforms generally had larger contributions of Bermuda Chub than other structure types. The deck site exhibited higher amounts of Vermilion Snapper than most other structures. Topples and cutoffs most differed in the contribution of Horse-eye Jack (higher on cutoffs). Topples also had higher contributions of Spotfin Hogfish than ships. Ships generally had stronger contributions of Gray Triggerfish and Sheepshead compared to other structures.

**Table 4:** Results from Analysis of Similarity pairwise comparisons.

	R	Significance	Highest Contributing	Second Highest Contributing
Groups	Statistic	Level	Species	Species
topple, cutoff	0.153	**0.012**	Horse-eye Jack (6.20%)—cutoff	Vermilion Snapper (4.89%)—cutoff
topple, ship	0.484	**0.002**	Spotfin Hogfish (5.73%)—topple	Blue Runner (5.60%)—ship
topple, deck	-0.252	0.967	—	—
topple, standing	0.302	**0.004**	Bermuda Chub (8.38%)—standing	Horse-eye Jack (4.79%)—standing
cutoff, ship	0.918	**0.001**	Creolefish (6.85%)—cutoff	Spotfin Hogfish (6.56%)—cutoff
cutoff, deck	0.379	**0.042**	Blue Runner (7.00%)—deck	Vermilion Snapper (6.10%)—deck
cutoff, standing	0.173	0.090	—	—
ship, deck	0.483	**0.036**	Vermillion Snapper (11.72%)—deck	Blue Runner (6.82%)—deck
ship, standing	0.91	**0.001**	Bermuda Chub (8.84%)—standing	Horse-eye Jack (5.48%)—standing
deck, standing	0.564	**0.048**	Vermillion Snapper (6.95%)—deck	Blue Runner (6.34%)—deck

For each comparison the top two species contributing most to the disparity is listed (via SIMPER) as well as the percent contribution.

**Fig 7:**
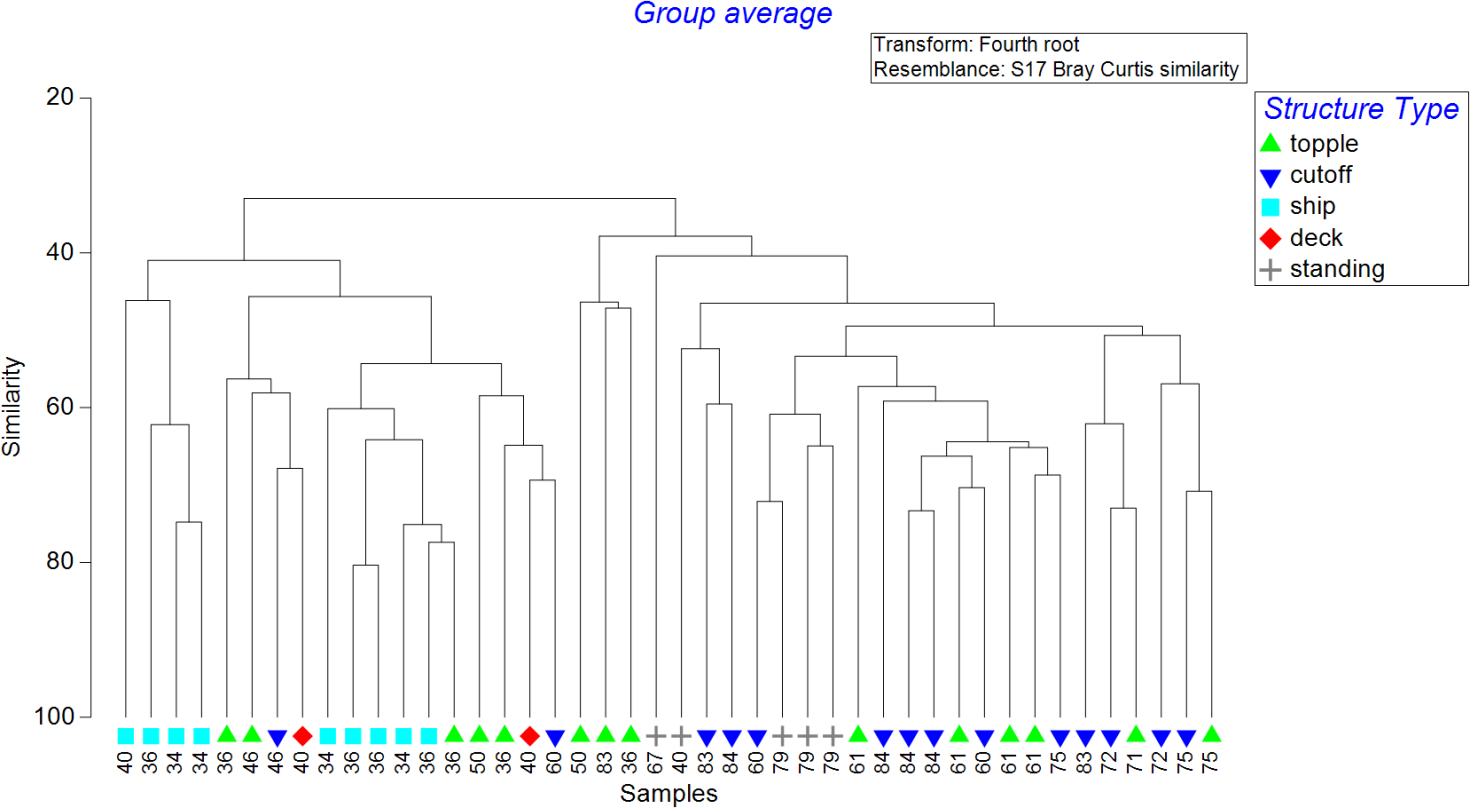
CLUSTER diagram showing linkages of assemblages by structure type and bottom depth. Linkages shown are based on Bray-Curtis similarity matrix. Values next to symbols represent bottom depths (meters).

Resemblance matrices between structure characteristics and the corresponding fish assemblage exhibited statistically significant agreement (RELATE test; ρ = 0.329; P = 0.001). The subsequent BIO-ENV test identified that ambient bottom depth alone produced the highest Spearman rank correlation value (ρ = 0.568; P = 0.001) among all possible combinations of the five factors tested (Table 5). A subsequent CLUSTER analysis indicated that the fish assemblages grouped into two main depth clusters (with exceptions): Cluster A (34–60 m), and Cluster B (88% of samples 60–84 m) (Fig 7). A subsequent SIMPER identified that the groups diverged via contributions of Horse-eye Jack, Creolefish, Vermilion Snapper, and Greater Amberjack, all of which were more prevalent in the deeper cluster.

**Table 5:** Results from BIO-ENV test examining the reef site characteristics best explaining fish assemblage composition.

Number of Variables	Spearman Correlation (ρ)	Vertical Relief	Shallowest Depth	Ambient Depth	Number of Structures
1	0.568			X	
2	0.515	X		X	
2	0.473		X	X	
3	0.467	X	X	X	
2	0.365	X	X		
3	0.343	X		X	X
4	0.329	X	X	X	X
2	0.328			X	X
1	0.320	X			
3	0.287		X	X	X

Various combinations of variables are listed with decreasing order of rho values.

### Red Snapper Vertical Longline Analyses

**Fig 8:**
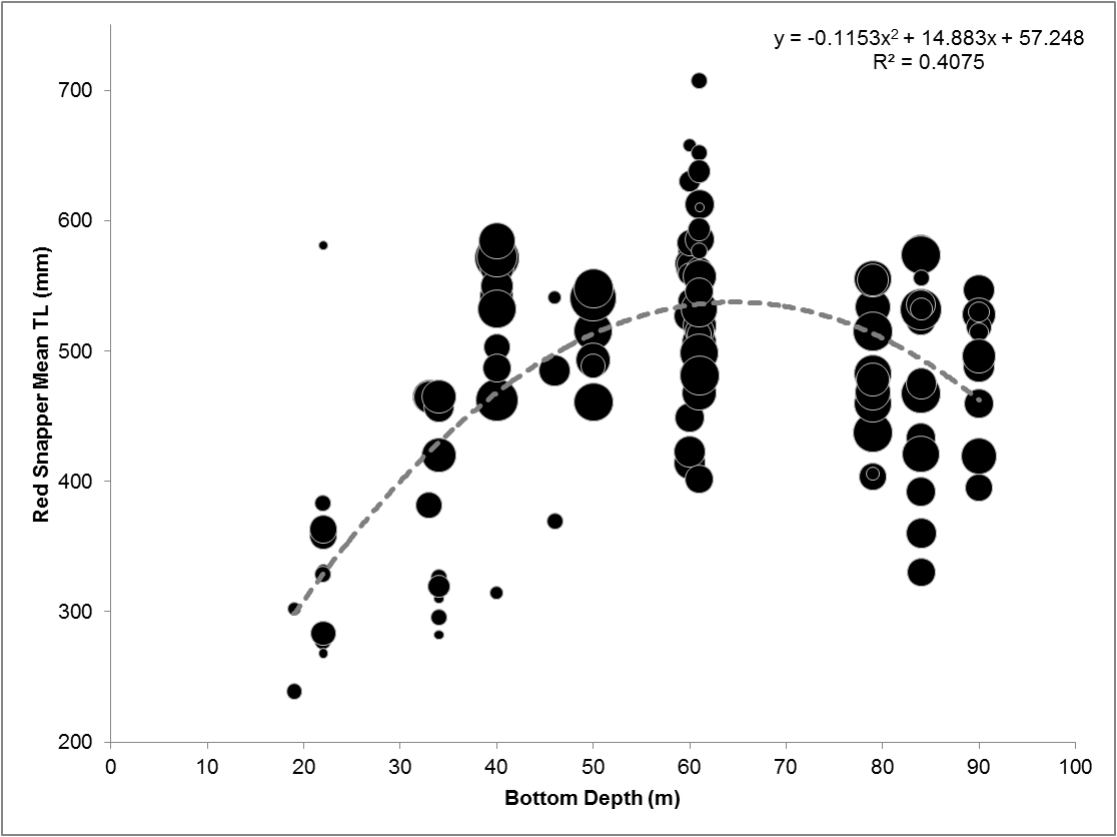
Bubble scatter plot of Red Snapper Mean Total Length (TL) by bottom depth. Circle size is scaled to the number of individuals contributing to the mean. A second order polynomial (quadratic) curve is fit to the data to demonstrate a potential non-linear relationship with bottom depth.

We found no statistical differences in Red Snapper CPUE among the different artificial structure types across our study sites (Kruskal-Wallis, H = 4.242; P = 0.374). However, structure type influenced Red Snapper Mean TL (ANOVA, *F*_4,111_ = 8.219; *P* < 0.001) and biomass (Kruskal-Wallis, *H* = 18.031; *P* = 0.001). Tukey’s and Dunn’s pairwise comparisons revealed that Red Snapper captured at Liberty Ships (mean = 388.5 mm TL; median biomass/set = 4.0 kg) were significantly smaller than fish caught on all other structure types. A subsequent scatter plot of mean TL by bottom depth indicated a quadratic-like distribution of Red Snapper size across depth (20–90 m), with smaller Red Snapper captured at the extremes of the depths sampled and the largest around 60 m depth (Fig 8).

## Discussion

Our broad-scale ROV survey documented fish communities over a range of artificial reef types in the western Gulf of Mexico. This video-based sampling approach captured a variety of species occupying these habitats from large mobile piscivores (e.g., sharks) to small herbivorous reef fishes. Further, we documented an array of economically important species (snappers, groupers, amberjacks, and pelagic sportfish) inhabiting the waters surrounding these reefs, further highlighting the economic potential of these artificial structures to enhance fisheries. Our ability to accurately document Red Snapper abundance using video-based methods was likely impaired by a persistent benthic nepheloid layer [41, 47]. As such, it is recommended that future monitoring of these sites include sampling with vertical longlines in conjunction with ROVs, or other means of indexing the abundance of this important species.

Our findings suggest that the conversion of standing platforms into permitted artificial reefs may significantly alter fish community structure, supporting previous gray literature [30, 47]. While overall levels of species richness, diversity, and evenness were stable among standing platforms and Rigs-to-Reefs (RTR) structures, our multivariate analyses documented significant key assemblage differences. The differences in fish communities at standing platforms was mainly driven by Bermuda Chub, a pelagic herbivore with low economic value. This species is likely more dominant at standing platforms due to greater availability of photosynthetic forage in the shallower portions of the water column [49–53]. Further, the surface features of the platform aggregate floating macroalgae such as *Sargassum* sp. (M.J. Ajemian, pers. obs), which may also serve as food, and/or transient habitat for Bermuda Chub. Importantly, while fish assemblages were different between standing and toppled platforms, we saw no significant differences between standing and cutoff platforms. This finding provides potential evidence that the community characteristics of standing platforms can be best retained by maintaining the upright orientation and relatively high vertical relief of these structures.

Our observations from a reefed platform deck (i.e., MI-712) suggest that this structure type could be highly productive material for fish communities. However, current U.S. Bureau of Safety and Environmental Enforcement platform removal policies prohibit their use as reefing material because of the difficulty of adequately removing hydrocarbons from their surfaces while at sea [54]. We found the highest average species richness at this structure type (Fig 3), and the highest counts of Gray and Vermilion Snapper in 2012 and 2013, respectively (Table 3). In fact, the Vermilion Snapper contribution differentiated the deck from several other structure types analyzed (Table 4). Given these potential diversity and fisheries benefits, which may be explained by the higher solid surface area and rugosity of platform decks, we strongly suggest exploration of more efficient cleaning methods and subsequent evaluation of these structures as artificial reef material in the Gulf of Mexico.

The effects of converting standing platforms into completely submerged reefs with lower relief are generally limited to pelagic planktivores and piscivores that use the upper water column, and do not affect important demersal species [29, 30]. We found no strong evidence of structure type affecting Red Snapper abundance (via ROV and vertical line surveys), biomass, or mean TL among RTR structures. However, further work is needed to investigate more subtle impacts of reefing options on other biological characteristics of Red Snapper (e.g., growth, fecundity, trophic position) and other broadly distributed fisheries species.

While bottom depth has been long recognized as playing an important role in demersal fish community structure in natural habitats [55–58] and is considered crucial to the success of artificial reefing programs [1], the impact of this factor on artificial reef fish assemblages is less known. Indeed, there is considerable knowledge of depth impacts on communities along standing platforms of the western Gulf of Mexico [26, 27, 59, 60], but the applicability of these trends to other artificial structure types (i.e., reefed platforms) has not been investigated. Previous studies of standing platforms have generally separated fish communities into 3 distinct groupings across the continental shelf – a “coastal” group (0–30 m), an “offshore” group (30–60 m), and a “bluewater” group (>60 m) [59]. Our cluster analysis of multiple artificial reef types supports this categorization derived by Gallaway et al. [59] as we observed a similar transition between fish assemblages from 34–60 m and 60–84 m along the Texas continental shelf. These differences were mainly driven by largely schooling species such Horse-eye Jack, Creolefish, and economically important Vermilion Snapper and Greater Amberjack, all of which were more prevalent in the deeper (or bluewater) cluster. While not apparent from multivariate analyses (likely due to low minimum counts), some species such as Sheepshead and Gray Triggerfish (both of which are recreationally or commercially exploited) were restricted to the shallower cluster. The higher contribution of Sheepshead along shallower artificial reefs is consistent with this species’ association with complex inshore and coastal habitats elsewhere [61, 62] and along nearshore platforms in the Gulf of Mexico [27, 63]. Gray Triggerfish are similarly reliant on the nearshore region out to 55 m [64, 65]. For species specific management purposes, low-relief, shallower sites may therefore be important for these more coastal species. Collectively, our data suggest that variation in artificial reef fish assemblages can be largely explained by trends in ambient natural communities, as demonstrated on small concrete artificial reefs off Florida [66]. Thus, resource managers should be mindful of this factor in future reefing efforts.

Our fishery-independent ROV and vertical line data from artificial reefs support previous literature documenting the broad shelf-wide distribution of Red Snapper [67]; this species was encountered across all sites and depths covered by our surveys. Importantly, our vertical line data also suggest that Red Snapper size may be maximized around depths of 60 m, at the interface of the offshore and bluewater assemblage zones. These data will be important when selecting reefing sites with the goal of enhancing Red Snapper productivity. It is currently not clear why such peaks are observed around such depths, although this trend may be related to the ambient—natural bank habitat that Red Snapper are known to use in this region [68, 69]. While CPUE had no clear association with depth from our vertical line surveys, bottom longline data from a previous study suggest the deeper portion of our sampling area (i.e., 60–90 m) supports relatively higher catch rates of Red Snapper in the Gulf of Mexico [70]. As such, expanded reefing efforts in this zone have the potential to improve the abundance of this important stock.

Vertical relief is often cited as a crucial factor in the design and success of artificial reefs [1, 71]. A previous study by Dokken et al. [47] recommended maximizing vertical relief to enhance diversity and richness of artificial reef biota. While further work is needed across structures with varying dimensions and a greater range of depths, our study also suggests that the vertical aspect of reefs may be important to fish species richness to a limit. We specifically observed richness levels saturate at reef heights of approximately 20 m off the bottom. Combined with the general management guideline of a minimum 26 m clearance for large vessel traffic in the Gulf of Mexico, similar to Dokken et al. [47] we suggest that reefing efforts in this region of the Gulf could be maximized along bottom depths of approximately 50 m with the use of vertically extensive RTR materials. We encourage reef placement at this depth, as it seems to represent a transition between offshore and blue water communities (see above), and may therefore bolster species richness. Given standard recreational dive limits of 39 m, reefing materials (i.e., cutoff platform jackets) at 50 m would also permit divers to interact with nearly 80% of the water column, and a wide array of species. Although such structures would be located at a considerable distance from shore, this was not previously found to be an important factor to sport divers off the Texas coast [72]. Thus, our data suggest that concentrating efforts at this depth has the potential to provide significant social and economic benefits. We would recommend additional socioeconomic studies to assess how the location of these reefs would have the most benefit.

### Conclusions

Our work indicates that reefing programs should carefully consider the ambient environmental conditions (i.e., depth), as these will most certainly affect fish assemblage composition and some characteristics of exploited fisheries species. Along the Texas continental shelf, our data show that reefing materials at 50–60 m bottom depth is most ideal and serves a dual purpose of both enhancing diving experiences as well as providing the best potential habitat for larger Red Snapper. Although not an important factor affecting our observations of fish assemblage composition, it is likely that the number of structures on a given reefing site may have considerable impacts on densities of certain species such as Red Snapper [35, 36, 67]. For example, further investigation into structure density effects is needed from this region as these materials are considerably larger than artificial reefs examined in previous studies. Additional factors that should be considered in future fish community assessments include reef age, proximity to natural habitat (e.g., south Texas hard banks), and fishing pressure. Finally, for exploited fisheries species we also recommend simultaneous fishery-independent comparisons between artificial and nearby natural reefs to better evaluate the stock-enhancing potential of these structures. Thus, while the ability of reefed platforms to act as surrogate reef fish habitat remains unresolved without these key analyses, our study clearly demonstrates that a variety of important species are associated with reefed platforms and Liberty Ships and therefore require continued monitoring efforts.

## References

[1] Baine M. Artificial reefs: a review of their design, application, management and performance. Ocean Coast Manage. 2001; 44: 241–259.

[2] Baine M. Side J. Habitat modification and manipulation as a management tool. Rev Fish Biol Fisher. 2003; 13: 187–199.

[3] Dupont JM. Artificial reefs as restoration tools: A case study on the west Florida Shelf. Coast Manage. 2008; 36: 495–507.

[4] Oh C, Ditton RB, Stoll JS. The economic value of scuba diving use of natural and artificial reef habitats. Soc Natur Resour. 2008; 21(6): 455–468.

[5] Pitcher TJ, Seaman W Jr. Petrarch’s Principle: how protected human-made reefs can help the reconstruction of fisheries and marine ecosystems. Fish Fish. 2000; 1(1): 73–81.

[6] Jorgensen D. An oasis in a watery desert? Discourses on an industrial ecosystem in the Gulf of Mexico Rigs‐to‐Reefs program. Hist Technol. 2009; 25(4): 343–364.

[7] Kaiser MJ. The Louisiana artificial reef program. Mar Policy. 2006; 30: 605–623.

[8] Love MS, Schroeder DM, Nishimoto MM. The ecological role of oil and gas production platforms and natural outcrops on fishes in southern and central California: a synthesis of information. Report to U. S. Department of the Interior, U. S. Geological Survey, Biological Resources Division, Seattle, WA. 2003; OCS Study MMS 2003–032.

[9] Dauterive L. Rigs to Reefs Policy, Progress, and Perspective. U.S. Department of the Interior, Minerals Management Service, Gulf of Mexico OCS Region, New Orleans, LA. 2000 Report. OCS Report MMS 2000–073.

[10] Gallaway BJ, Szedlmayer ST, Gazey WJ. A life history review for red snapper in the Gulf of Mexico with an evaluation of the importance of offshore petroleum platforms and other artificial reefs. Rev Fish Sci. 2009; 17: 48–67.

[11] Lindquist DC, Shaw R, and Hernandez FJ Jr. Distribution patterns of larval and juvenile fishes at offshore petroleum platforms in the north-central Gulf of Mexico. Estuar Coast Shelf S. 2005; 62: 655–665.

[12] Simmons CM, Szedelmeyer ST. Recruitment of Age-0 Gray Triggerfish to benthic structured habitat in the northern Gulf of Mexico. T Am Fish Soc. 2011; 140: 14–20.

[13] Szedlmayer ST, Shipp RL. Movement and growth of Red Snapper, Lutjanus campechanus, from an artificial reef area in the northeastern Gulf of Mexico. Bull Mar Sci. 1994; 55: 887–896.

[14] Polovina JJ. Fisheries applications and biological impacts of artificial habitats. In: Seaman W, editor. Artificial Habitats for Marine and Freshwater Fisheries. 1991. pp. 153–176.

[15] Reubens JT, Braeckman U, Vanaverbeke J, Van Colen C, Degraer S, Vincx M. Aggregation at windmill artificial reefs: CPUE of Atlantic cod (Gadus morhua) and pouting (Trisopterus luscus) at different habitats in the Belgian part for the North Sea. Fish Res. 2013; 139: 28–34.

[16] Zalmon IR, Novelli R, Gomes MP, Faria VV. Experimental results of an artificial reef programme on the Brazilian coast north of Rio de Janeiro. ICES J Mar Sci. 2002; 59: S83–S87.

[17] Goodsell PJ, Chapman MG. Rehabiliation of habitat and the value of artificial reefs. In: Marine Hard Bottom Communities. Wahl M, editor. Ecol Stu. 2009; 206: 333–344.

[18] Grossman GD, Jone GP, Seaman WJ Jr. Do artificial reefs increase regional fish production? A review of existing data. Fisheries. Special issue on artificial reef management. 1997; 22(4): 17–23.

[19] Shipp RL, Bortone SA. A prospective of the importance of artificial habitat on the management of red snapper in the Gulf of Mexico. Rev Fish Sci. 2009; 17: 41–47.

[20] Love MS, Schroeder DM, Lenarz WH. Distribution of Bocaccio (Sebastes paucispinis) and cowcod (Sebastes levis) around oil platforms and natural outcrops off California with implications for larval production. B Mar Sci. 2005; 77(3): 397–408.

[21] Love MS, Schroeder DM, Lenarz W, MacCall A, Bull AS, Thorsteinson L. Potential use of offshore marine structures in rebuilding an overfished rockfish species, bocaccio (Sebastes paucispinis). Fish Bull. 2006; 104: 383–390.

[22] Love MS, York A. The relationships between fish assemblages and the amount of bottom horizontal beam exposed at California oil platforms: fish habitat preferences at man-made platforms and (by inference) at natural reefs. Fish Bull. 2006; 104: 542–549.

[23] Claisse JT, Pondella DJ II, Love M, Zahn LA, Williams CM, Williams JP, et al Oil Platforms off California are among the most productive marine fish habitats globally. P Natl Acad Sci USA. 2014; 111(43): 15462–67. 10.1073/pnas.1411477111 25313050 PMC4217422

[24] Cresson P, Ruitton S, Harmelin-Vivien M. Artificial reefs do increase secondary biomass production: mechanisms evidenced by stable isotopes. Mar Ecol Prog Ser. 2014; 509: 15–26.

[25] Stanley DR, Wilson CA. Factors affecting the abundance of selected fishes near oil and gas platforms in the northern Gulf of Mexico. Fish Bull. 1991; 89: 149–159.

[26] Stanley DR, Wilson CA. Abundance of fishes associated with a petroleum platform as measured with dual-beam hydroacoustics. ICES J Mar Sci. 1996; 53: 473–475.

[27] Stanley DR, Wilson CA. Seasonal and spatial variation in the abundance and size distribution of fishes associated with a petroleum platform in the northern Gulf of Mexico. Can J Fish Aquat Sci. 1997; 54(5): 1166–1176.

[28] Stanley DR, Wilson CA. Variation in the density and species composition of fishes associated with three petroleum platforms using dual beam hydroacoustics. Fish Res. 2000; 47: 161–172.

[29] Wilson CA, Miller MW, Allen YC, Boswell KM, Nieland DL. Effects of depth, location, and habitat type on relative abundance and species composition of fishes associated with petroleum platforms and Sonnier Bank in the Northern Gulf of Mexico. Report to U.S. Department of Interior, Minerals Management Service, Gulf of Mexico OCS Region, New Orleans, LA. 2006; OCS Study MMS 2006–037.

[30] Wilson CA, Pierce A, Miller MW. Rigs and Reefs: A comparison of the fish communities at two artificial reefs, a production platform, and a natural reef in the Northern Gulf of Mexico. Report to U.S. Department of Interior, Minerals Management Service, Gulf of Mexico OCS Region, New Orleans, LA. 2003; OCS Study MMS 2003–009.

[31] Rooker JR, Dokken QR, Pattengill CV, Holt GJ. Fish assemblages on artificial and natural reefs in the Flower Garden Banks National Marine Sanctuary, USA. Coral Reefs. 1997; 16: 83–92.

[32] Schroepfer R, Szedlmayer ST. Estimates of residence and site fidelity for Red Snapper (Lutjanus campechanus) on artificial reefs in the northeastern Gulf of Mexico. Bull Mar Sci. 2006; (1): 93–101.

[33] Szedlmayer ST, Schroepfer RL. Long-term residence of Red Snapper on artificial reefs in the northeastern Gulf of Mexico. T Am Fish Soc. 2005; 134(2): 315–325.

[34] Pulsipher AG, Iledare OO, Mesyanzhinov DV, Dupont A, Zhu QL. Forecasting the number of offshore platforms on the Gulf of Mexico OCS to the Year 2023. Report to U.S. Department of the Interior, Minerals Management Service, Gulf of Mexico OCS Region, New Orleans, LA. 2001; OCS Study MMS 2001–013.

[35] Strelcheck AJ, Cowan JH, Shah A. Influence of reef location on artificial reef fish assemblages in the northcentral Gulf of Mexico. Bull Mar Sci. 2005; 77(3): 425–440.

[36] Mudrak PA, Szedlmayer ST. Proximity effects of larger resident fishes on recruitment of age-0 Red Snapper in the Northern Gulf of Mexico. T Am Fish Soc. 2012; 141: 487–494.

[37] Jorgensen D. OSPAR’s exclusion of rigs-to-reefs in the North Sea. Ocean Coast Manage. 2012; 58: 57–61.

[38] Jorgensen D. Rigs-to-reefs is more than rigs and reefs. Peer reviewed letter. Front Ecol Environ. 2012; 10.1890/12.WB.012

[39] Macreadie PI, Fowler AM, Booth DJ. Rigs-to-reefs: will the deep sea benefit from artificial habitat? Front Ecol Environ. 2011; 9: 455–461.

[40] Macreadie PI, Fowler AM, Booth DJ. Rigs-to-reefs policy: can science trump public sentiment? Peer reviewed letter. Front Ecol Environ. 2012; 10.1890/12.WB.012.2012

[41] Ajemian MJ, Wetz JJ, Shipley-Lozano B, Stunz GW. Rapid assessment of fish communities on submerged oil and gas platform reefs using remotely operated vehicles. Fish Res. 2015; 167: 143–155.

[42] Ellis DM, DeMartini EE. Evaluation of a video camera technique for indexing abundances of juvenile pink snapper, Pristipomoides filamentosus, and other Hawaiian insular shelf fishes. Fish Bull. 1995; 93: 67–77.

[43] Merritt D, Parke M, Donovan MK, Wong K, Kelley C, Drazen JC, et al BotCam: a baited camera system for nonextractive monitoring of bottomfish species. Fish Bull. 2011; 109: 56–67.

[44] Watson DL, Harvey ES, Anderson MJ, Kendrick GA. A comparison on temperate reef fish assemblages recorded by three underwater stereo-video techniques. Mar Biol. 2005; 148: 415–425.

[45] Willis TJ, Millar RB, Babcock RC. Detection of spatial variability in relative density of fishes: comparison of visual census, angling, and baited underwater video. Mar Ecol Prog Ser. 2000; 198: 249–260.

[46] Clarke KR, Gorley RN. PRIMER v6: User manual/tutorial PRIMER-E, Plymouth, UK 2006.

[47] Dokken QR, Withers K, Childs S, Riggs T. Characterization and comparison of platform reef communities off the Texas coast. 2000 Final Report to Texas Parks and Wildlife Artificial Reef Program. TAMU-CC-0007-CCS.

[48] Gregalis KC, Schlenker LS, Drymon JM, Mareska JF, Powers SP. Evaluating the performance of vertical longlines to survey reef fish populations in the Northern Gulf of Mexico. T Am Fish Soc. 2012; 141: 1453–1464.

[49] Downie RA, Babcock RC, Thomson DP, Vanderklift MA. Density of herbivorous fish and intensity of herbivory are influenced by proximity to coral reefs. Mar Ecol Prog Ser. 2013; 482: 217–225.

[50] Michael PJ, Hyndes GA, Vanderklift MA, Verges A. Identity and behavior of herbivorous fish influence large-scale spatial patterns of macroalgal herbivory in a coral reef. Mar Ecol Prog Ser. 2013; 482:227–240.

[51] Moore D. Development, distribution, and comparison of rudder fishes Kyphosus sectatrix (Linnaeus) and K. incisor (Cuvier) in the western North Atlantic. Fish Bull. 1962; 61: 451–480.

[52] Randall JE. An analysis of the fish populations of artificial and natural reefs in the Virgin Islands. Caribb J Sci. 1963; 3(1): 31–47.

[53] Silvano RAM, Güth AZ. Diet and feeding behavior of Kyphosus spp. (Kyphosidae) in a Brazilian subtropical reef. Braz Arch Biol Techn. 2006; 49(4): 623–629.

[54] U.S. Minerals Management Service. Rigs-to-Reefs Policy Addendum: Enhanced Reviewing and Approval Guidelines in Response to the Post-Hurricane Katrina Regulatory Environment. U.S. Department of Interior, Gulf of Mexico OCS Region, New Orleans, LA 12 31, 2009.

[55] Bell JD. Effects of depth and marine reserve fishing restrictions on the structure of a rocky reef assemblage in the north-western Mediterranean Sea. J Appl Ecol. 1983; 20: 357–369.

[56] Garcia-Sais JR. Reef habitats and associated sessile-benthic and fish assemblages across a euphotic–mesophotic depth gradient in Isla Desecheo, Puerto Rico. Coral Reefs. 2010; 29: 277–288.

[57] McClatchie S, Millar RB, Webster F, Lester PJ, Hurst R, Bagley N. Demersal fish community diversity off New Zealand: Is it related to depth, latitude and regional surface phytoplankton? Deep-Sea Res PT I. 1997; 44(4): 647–667.

[58] Zintzen V, Anderson MJ, Roberts CD, Harvey ES, Stewart AL, Struthers CD. Diversity and Composition of Demersal Fishes along a Depth Gradient Assessed by Baited Remote Underwater Stereo-Video. Plos Biol. 2012; 7(10): e48522 10.1371/journal.pone.0048522 PMC3485343 23119045

[59] Gallaway BJ, Johnson MF, Martin LR, Margraf FJ, Lewbel GS, Howard RL, et al. The artificial reef studies. Vol. 2. In: Bedinger Jr CA, Kirby LZ, editors. Ecological investigations of petroleum production platforms in the central Gulf of Mexico. Bureau of Land Management, New Orleans OCS, LA. 1981; SWKI Project 01–5245.

[60] Gallaway BJ, Lewbel GS. The ecology of petroleum platforms in the northwestern Gulf of Mexico: A community profile. U.S. Fish and Wildlife Service, Office of Biological Services, Washington, D.C. FWS/OBS-82/27. Bureau of Land Management, Gulf of Mexico OCS Regional Office. 1982; Open-File Report 82–03.

[61] Parker RO Jr, Chester AJ, Nelson RS. A video transect method for estimating reef fish abundance, composition, and habitat utilization at Gray’s Reef National Marine Sanctuary, Georgia. Fish Bull. 1994; 92(4): 787–799.

[62] Steimle FW, Zetlin C. 2000. Reef Habitats in the Middle Atlantic Bight:Abundance, Distribution, Associated Biological Communities, and Fishery Resource Use. Mar Fish Rev. 2000; 62(2): 24–42.

[63] Gallaway BJ, MF Johnson, GS Boland, GS Lewbel, LR Martin, FJ Margraf, et al. The artificial reef studies. In: Ecological investigations of petroleum production platforms in the central Gulf of Mexico. 1979 Final report to Southwest Research Institute for the Bureau of Land Management. Washington, D.C.

[64] Hanisko D, Rester J. Gray Triggerfish In: Gulf of Mexico Data Atlas [Internet]. Stennis Space Center (MS): National Coastal Data Development Center; 2012 [3 screens]. Available: http://gulfatlas.noaa.gov/

[65] McEachran JD, Feldheim JD. Fishes of the Gulf of Mexico, Vol. 2, Scorpaeniformes to Tertraodontiformes, University of Texas Press, Austin 1998.; 1004 p.

[66] Sherman RL, Gilliam DS, Spieler, RE. A preliminary examination of depth associated spatial variation in fish assemblages on small artificial reefs. J Appl Ichthyol. 1999; 15: 116–121.

[67] Gallaway BJ, Cole JG, Meyer R, Roscigno P. Delineation of essential habitat for juvenile Red Snapper in the northwestern Gulf of Mexico. T Am Fish Soc. 1999; 128: 713–726.

[68] Dennis GD, Bright TJ. Reef fish assemblages on hard banks in the northwestern Gulf of Mexico. B Mar Sci. 1988; 43: 280–307.

[69] Rezak R, Gittings SR, Bright TJ. Biotic assemblages and ecological controls on reefs and banks of the northwest Gulf of Mexico. Am Zool. 1990; 30: 25–35.

[70] Mitchell KM, Henwood T, Fitzhugh GR, Allman RJ. Distribution, abundance, and age structure of red snapper (Lutjanus campechanus) caught on research longlines in U.S. Gulf of Mexico. Gulf of Mexico Science. 2004; 2: 164–172.

[71] Rilov G, Benayahu Y. Fish assemblage on natural versus vertical artificial reefs: the rehabilitation perspective. Mar Biol. 2000; 136: 931–942.

[72] Ditton RB, Osburn HR, Baker TL, Thailing CE. Demographics, attitudes, and reef management preferences of sport divers in offshore Texas waters. ICES J Mar Sci. 2002; 59: S186–S191.

